# Prevention of Severe Vestibular Hypofunction after Systemic Gentamicin

**DOI:** 10.3390/jcm11030586

**Published:** 2022-01-25

**Authors:** Sofía Ferreira-Cendon, Ramon Martinez-Carranza, Maria José Fernandez-Nava, Rosana Villaoslada-Fuente, Hortensia Sanchez-Gomez, Santiago Santa Cruz-Ruiz, María Sanchez-Ledesma, Angel Batuecas-Caletrio

**Affiliations:** 1 Neurotology Unit, ENT Department, University Hospital of Salamanca, IBSAL, 37007 Salamanca, Spain; sferreira@saludcastillayleon.es (S.F.-C.); ramcmed07@gmail.com (R.M.-C.); mjfnava@saludcastillayleon.es (M.J.F.-N.); rvillaoslada@saludcastillayleon.es (R.V.-F.); hortensiasanchez@saludcastillayleon.es (H.S.-G.); santaorl@usal.es (S.S.C.-R.); 2 Neurotology Unit, ENT Department, Faculty of Medicine, University of Salamanca, 37007 Salamanca, Spain; 3 Department of Internal Medicine, Infectious Diseases, University Hospital of Salamanca, IBSAL, 37007 Salamanca, Spain; mledesma@saludcastillayleon.es

**Keywords:** systemic gentamicin, infective endocarditis, vestibular hypofunction, oscillopsia, video head impulse test

## Abstract

The importance of early evaluation by a neurotologist in patients with infective endocarditis treated with systemic gentamicin and its impact on the patients’ quality of life was evaluated. This is a longitudinal retrospective cohort study of 29 patients who received intravenous gentamicin for the treatment of infective endocarditis. Patients were classified into two groups: group A, before a neurotologist was included in the treatment protocol, and group B, after the inclusion of a neurotologist. The frequency of the different symptoms in each group was measured, and the gain of the vestibulo-ocular reflex (VOR) and its relationship with the presence of oscillopsia. In total, 13 and 16 patients were assigned to groups A and B, respectively. The mean gain of the VOR measured using the video head impulse test in group A was 0.44 in the best side and 0.39 in the worst side. In group B, the mean gain was 0.71 (best side) and 0.64 (worst side) (*p* < 0.0001). The patients who complained about oscillopsia had a main gain of 0.41 in the best side and 0.35 in the worst side. Evaluation of vestibular function should be included in the infective endocarditis treatment protocol, including the adverse effects of systemic gentamicin.

## 1. Introduction

Aminoglycosides are bactericidal antimicrobial agents that disrupt the integrity of the bacterial cell wall and impair bacterial protein synthesis [1]. These antibiotics are most frequently used to treat life-threatening infections and as synergistic agents together with other antibiotics for the treatment of infective endocarditis [2], especially gentamicin.

The use of aminoglycosides is limited by their side effects, including ototoxicity, which is manifested by the dysfunction of the auditory (hearing loss) or vestibular (balance deficit) system. Other remarkable side effects include nephrotoxicity and neuromuscular blockade [3].

Aminoglycoside-induced ototoxicity has been linked to genetic susceptibility. Some studies identified the A1555G mutation in the mitochondrial 12s rRNA gene as a primary genetic factor in cases of aminoglycoside-induced ototoxicity [4].

Some aminoglycosides affect hearing more, whereas others are more harmful to the vestibular system, including gentamicin [5]. Some authors suggest that gentamicin vestibulotoxicity is not related to elevated serum levels of the drug (it does not depend on the dosage), which means that it is idiosyncratic [6]. This antibiotic produces bilateral vestibular loss without vertigo [7]. Although it is usually bilateral and symmetric, it can also be unilateral [8].

Otherwise, there are well-established factors that predispose individuals to vestibulotoxicity, for example, prolonged pharmacological therapy and some patient-related factors (e.g., renal insufficiency, increasing age, and concomitant use of other ototoxic drugs) [9,10].

The vestibulotoxic effect of gentamicin involves immediate inhibition of hair cell transmitter release by blocking mechanotransduction [11], and sustained exposure causes vestibular hair cell damage and death due to apoptosis. Type I hair cells are more susceptible to loss than type II hair cells because they show an increased uptake and retention of gentamicin [12].

The impairment of cellular activity due to aminoglycosides may be temporary or permanent [13]. Early recognition of impending vestibulotoxicity is very important to the prevention of permanent harm because bilateral vestibular loss profoundly affects an individual’s quality of life [5].

Vestibulotoxicity is suspected if a patient complains of imbalance, which is worse while walking in the dark, and oscillopsia during head movements [14]. Vertigo is infrequent [15]. Oscillopsia is a disabling condition in patients with bilateral vestibular hypofunction (BVH). When the vestibulo-ocular reflex (VOR) is bilaterally impaired, its ability to compensate for rapid head movements must be supported by refixation saccades [16].

The video head impulse test (vHIT) is a rapid physiological test that can be used to quantify vestibular function. It identifies unilateral or bilateral vestibular weakness [17]. Currently, it is one of the most used diagnostic options, and its utility in the field of neurotology has been invaluable. It is a quick and easy to perform test to evaluate vestibular function.

In many cases, secondary symptoms (such as instability) that may present as a side effect of this drug are not taken into account due to the severity of the infective endocarditis. Patients treated with aminoglycosides are often critically ill and bedridden. Thus, there may be a considerable delay in diagnosing bilateral vestibulopathy in these cases without systematic evaluation. Knowing that gentamicin can cause a vestibular deficit and that a simple test can detect this deficit, it could be very useful to include a neurotologist in the treatment team for patients with infective endocarditis.

The aim of this study was to clarify the importance of an early evaluation by a neurotologist in patients with infective endocarditis treated with systemic gentamicin in the development of vestibular toxicity and its impact on the patients’ quality of life.

## 2. Materials and Methods

This was a longitudinal retrospective cohort study of 29 patients who were hospitalized in the cardiology service of a tertiary referral hospital and received systemic gentamicin for the treatment of infective endocarditis. Patients were classified into two groups: group A comprised patients who received treatment before 2017, that is, before a neurotologist was included in the treatment and follow-up protocol for patients with infective endocarditis, and group B comprised patients who received treatment between January 2017 and December 2019.

In group A, patients received treatment without control or exhaustive monitoring by a neurotologist. These patients sought consult at the outpatient clinic referred by a cardiologist due to intense and permanent vestibular symptoms after the discontinuation of treatment. In group B, patients underwent exhaustive monitoring by a neurotologist at the symptom onset.

During the monitoring process, the patient was questioned every day about the appearance of vestibular symptoms, dizziness, or imbalance. When any of the patients, throughout the treatment, reported the appearance of any of these symptoms, they were evaluated by the neurotologist and gentamicin was withdrawn from the patient’s treatment. In this evaluation, in addition to an exhaustive neurotological examination (including the clinical head impulse test), an audiometry and a video head impulse test were performed.

The patient was re-evaluated before discharge and one month after discharge.

The infective endocarditis protocol in this referral hospital (prior to confirmation of an adequate kidney function) is to start with an empirical treatment (cloxacillin 12 g/24 h IV + ampicillin 12 g/24 h IV + gentamicin 3 mg/kg/24 h IV). The neurotologist’s role was to monitor patients who, during their hospital stay, began to experience balance-related symptoms, such as dizziness, instability, or oscillopsia. In the outpatient clinic, patients were interviewed and vHIT was performed. Subsequently, the cardiologist and neurotologist evaluated the risk/benefit of gentamicin discontinuation and opted for the second-line antimicrobial option to prevent further progression of vestibulotoxicity.

After a month, a second vHIT was performed in group B. Since patients in group A were not evaluated by the neurotologist during their hospital stay, only the vestibular function at the time of the neurotology outpatient clinic visit was evaluated, and it was not possible to assess their vestibular function at follow-up. Patients with previous vestibular disorders were not included in the study.

For the vHIT, we used the ICS Impulse^®^ device, with software version 4.0 (Otometrics A/S, Taastrup, Denmark), consisting of lightweight tight-fitting goggles with an embedded accelerometer and a 250 Hz sampling rate video camera that captures the patient’s right eye movements, as reflected on a half-silvered mirror aided by a low-level infrared light-emitting diode. The distance from the target to the pupil was 1 m, and calibration was performed before the vestibulo-ocular testing procedure was initiated. In total, 40 impulses were delivered (20 in each direction), randomizing the intervals and testing side. The minimum accepted peak speed was 150 deg/s. Vertical impulses were not considered in the present study.

To evaluate the results, the absolute mean VOR gain was considered. The gain of the VOR was obtained after each head impulse and calculated as the ratio of the eye velocity to the head velocity; the procedure in our system measures the area under the curve ratio of the head velocity and eye velocity. The mean of the different impulses in each direction is given but as gain referenced to the affected (mean VOR gain for ipsilesional) or non-affected (contralesional) side. Normal gain is defined as ≥0.80 and abnormal when <0.80. The relative gain value was calculated as the amount of gain asymmetry according to the formula: Gas = (1 − (lower gain/higher gain)) × 100 (%) [18].

BVH consists of a bilaterally reduced or absent angular VOR function (which is documented by a bilaterally pathological horizontal angular VOR gain of <0.6, as measured by the vHIT). It has been included in the diagnostic criteria for bilateral vestibulopathy in the consensus document of the Classification Committee of the Bárány Society [19].

The patients were specifically asked about their oscillopsia. These questions were presented as “yes or no” questions. For an affirmative answer, the patient had to mention permanent and non-fluctuating oscillopsia while walking through different environments in their daily life. During the anamnesis, the following questions about oscillopsia in daily life activities were used: Does the world around you seem to move or jump when you are sitting, standing, or walking? Do you feel this sensation when you are running or driving? Is this sensation worse when walking on sand or grass? Can you read a poster while walking? Can you look for contacts in your mobile device while walking? Oscillopsia was considered to be present when the answers for all these questions were “Yes” [16].

A complete anamnesis and clinical examination, including an ocular-motor examination, was performed in all patients (spontaneous nystagmus, position nystagmus, head shaking nystagmus, saccades, smooth pursuit, and test of skew), and vestibular loss was confirmed using vHIT (gain < 0.8).

Statistical analysis was performed using SPSS Statistics 23 program. The Mann–Whitney U test (Bonferroni correction) was performed to compare the number of days that passed from the start of the treatment to the onset of symptoms until the ENT evaluation, the distribution of gains in the vHIT tests of both groups, and the relationship between the gains and the presence of oscillopsia. The Pearson’s chi-square test was performed to compare the frequencies of symptoms and oscillopsia in both groups.

The study was performed in accordance with the ethical guidelines of the 1975 Declaration of Helsinki. The study was approved by the institutional IRB (PI9810/2017A), and informed consent was obtained from each study participant.

## 3. Results

In the study, 29 patients were included, all of whom received intravenous treatment with gentamicin at a dose of 3 mg/kg/24 h. Between January 2010 and December 2016, 250 patients were treated in our hospital for infective endocarditis. Of them, 13 (5.2%) were referred to our outpatient clinic for dizziness or permanent instability. These 13 patients were included in group A. From January 2017 to December 2019, 79 patients were treated for infective endocarditis. Sixteen (20.2%) of these patients manifested with dizziness or imbalance after the initiation of treatment, and they were included in group B. 

The ages were 58 ± 6 years (group A) and 54 ± 7 years (group B). Seven men and six women were included in group A, and nine men and seven women in group B. No differences were observed in terms of age and sex (Table 1).

The aim of this study was to analyze the average time from treatment prescription to the neurotologist’s examination. The mean number of days from the beginning of the systemic gentamicin treatment to the assessment by the neurotologist was 70 days in group A compared to 6 days in group B (*p* < 0.0001).

Another measurement parameter was the timeframe at which the vestibular tests were performed after the symptoms started. The mean number of days from the onset of the symptoms (dizziness, imbalance, or oscillopsia) until the vestibular tests were performed was 65 days in group A and 2 days in group B (*p* < 0.0001).

When calculating the mean time from the initial prescription of the treatment to the onset of vestibular symptoms (recorded in the clinical history), both groups showed similar data: the time difference between the day when treatment started and the day when symptoms started was 5 days in group A and 4 days in group B (*p* = 0.076).

Considering the severity of the symptoms, 31% of the patients in group A showed only an imbalance, and 69% showed an imbalance and dizziness. Conversely, 69% of the patients in group B showed only an imbalance, and 31% suffered from an imbalance and dizziness (Table 1).

Oscillopsia is one of the most disabling symptoms in patients with BVH. In group A, 62% (8/13) of the patients suffered from oscillopsia during the neurotologist visit while in group B, only 19% (3/16) manifested it (*p* = 0.027) (Table 1).

A reduced vestibular test response is expected in both ears in cases of BVH. For this reason, we stratified the results of the vHIT as the “best side” or “worst side”.

**Figure 1:**
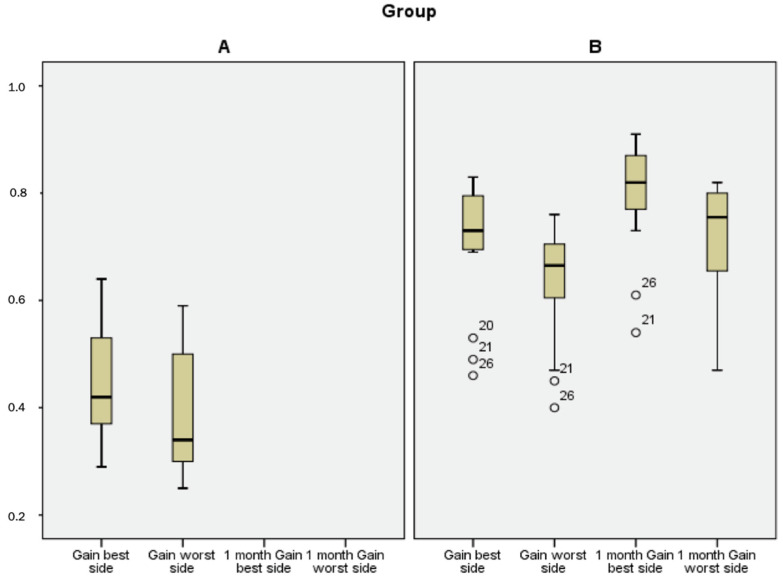
Box plot of the gains to the best or the worst side in each group of patients. (**A**): Group A at the first evaluation. (**B**): Group B at the first evaluation and after 1 month.

The mean gain to the worst side was 0.39 ± 0.11 in group A and 0.64 ± 0.11 in group B (*p* < 0.0001). The mean gain to the best side was 0.44 ± 0.10 in group A and 0.71 ± 0.12 in group B (*p* < 0.0001) (Figure 1).

After 1 month, the mean gain measured with the vHIT in group B was 0.72 ± 0.10 and 0.80 ± 0.10, respectively (Figure 1). None of the patients complained of oscillopsia at theone-month follow-up.

**Figure 2:**
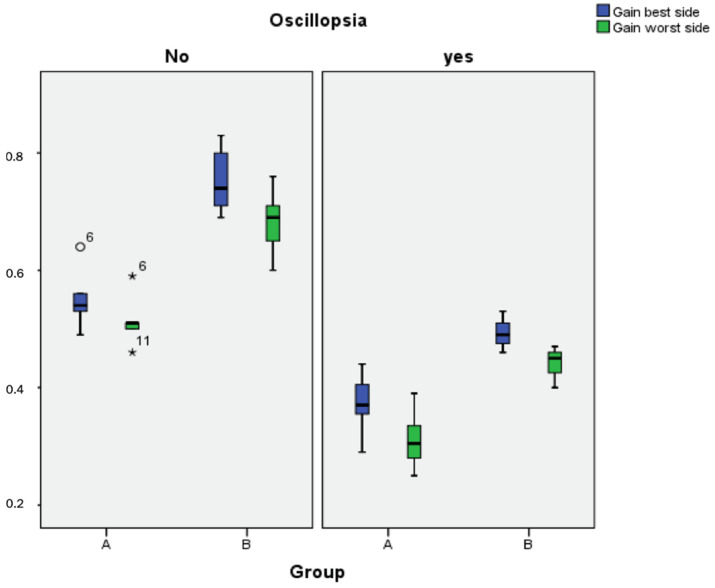
Patient gains and the presence of oscillopsia. Regardless of the group (A or B), the presence of oscillopsia is related to the low gain of the vestibulo-ocular reflex (* Atypcal values).

Another parameter calculated was the gain on both sides in patients with oscillopsia. The mean gain in patients (both groups) who showed oscillopsia was 0.41 in the best side and 0.35 in the worst side. The mean gain in patients who did not present oscillopsia was 0.70 in the best and 0.63 in the worst side, respectively (*p* < 0.0001) (Figure 2).

If we compare the patients in group A with the worst patients (patients with the worst vestibular function) in group B (the worst 20% of the patients), it is observed that the mean gain in group A was 0.39 while in the worst patients in group B, it was 0.44. It is necessary to highlight that the worst patients of group B, during their follow-up, improved their gains up to a mean gain of 0.55.

## 4. Discussion

It is necessary to begin this discussion by addressing the main limitation of the study. Two groups were compared in which the intervention was not identical. In the first one, there was no neurotological care during the admission of the patients, nor were they questioned about symptoms related to their balance until, due to their persistence, they were referred for evaluation in the neurotology unit. Conversely, the patients in the second group were monitored for their symptoms, and they were immediately treated by a neurotologist at the onset of symptoms. This may have resulted in a significant selection bias. 

However, the intention of this study was to investigate the importance of monitoring these symptoms in patients who were treated with systemic gentamicin for infective endocarditis. The results showed that this intervention is crucial. In fact, the difference between the days to be evaluated for instability, dizziness, or oscillopsia in both groups was more than 2 months (65 days vs. 2 days). Not asking about a certain symptom does not mean that patients do not suffer from it. In our opinion, asking about dizziness, instability, or oscillopsia in patients receiving systemic gentamicin (especially in long treatment regimens) should be recommended [14].

Trying to be even more rigorous, we compared the patients in group A (we understand that this group includes the most severe patients and those with the greatest symptoms in that series) with those patients in group B with more severe bilateral vestibular hypofunction. Not only did the worse patients in group B show less impairment in their vestibular function, but they also improved during the one-month follow-up.

There are very few references in the literature about the importance of the participation and early intervention of a neurotologist in the progression of vestibulotoxicity produced by systemic gentamicin [8,14] and none related to the treatment of infective endocarditis.

Bilateral vestibular loss after severe vestibulotoxicity implies a significant decrease in the patient’s quality of life [5]. Gentamicin can produce mild and early vestibulotoxicity (reversible and asymptomatic) in some patients [20]. Because of this, informing patients that the slightest symptom of imbalance should be reported and recommending routine vestibular testing are key to identifying vestibulotoxicity early and making a significant difference in the prognosis and development of permanent vestibular damage [8].

Vestibular symptoms are usually the initial adverse effect of systemic gentamicin therapy. Although physicians are well aware of this, these side effects are not readily recognized (especially vestibular symptoms), and these etiologies are under-reported [21]. In our study, groups A and B were compared, and there was a statistically significant difference between the mean number of days from the beginning of gentamicin therapy until the neurotologist’s evaluation of the patient. In the same way, this difference can also be seen from the day the symptoms started to the day when the evaluation was performed.

In total, 20% of the patients in group B were evaluated. In group A, 5% were evaluated. It is assumed that in group A, only those patients who showed more severe symptoms were evaluated and that some patients with a mild (or moderate) vestibular deficit were not evaluated. Good monitoring of these patients makes it possible to detect vestibular deficits before they become severe, with a greater alteration in the quality of life.

There was no statistically significant difference between the groups in terms of the time period between the start of treatment and the onset of symptoms. Symptoms are usually mild at the beginning, which is why patients do not report them until they are severely affected; however, vestibulotoxicity occurs very quickly. A study reported that all affected patients (treated with gentamicin) were asymptomatic but presented vestibular abnormality within 3.5 h after administration of gentamicin [8].

Symptoms were more severe in group A because there was a higher percentage of imbalances, dizziness, and oscillopsia. This group was more clinically affected when compared to group B. BVH is related to the total amount of gentamicin received, which can cause great impairment in an affected individual [5], and permanent damage results in persistent oscillopsia and disequilibrium [22].

Notably, 20% of the treated patients in group B were evaluated by a neurotologist. This percentage may seem high compared to other studies [8], but some of them did not present altered vestibular function. They were studied, and therefore, were included in the study because they presented some symptoms, generally only instability. It should also be considered that our patients receiving gentamicin were evaluated after a mean use of gentamicin of four days while other studies have assessed vestibular damage in patients in whom the use of gentamicin is necessary for one or two days [8].

The vHIT gain of both sides in group A was considerably lower than that in group B, and the gain of the best side in group A was lower than that of the worst side in group B. In another study, patients with gentamicin vestibular toxicity (GVT) showed a continuous spectrum of VOR gain deficits from almost normal to total bilateral vestibular loss (BVL) but received gentamicin for 1.9 days. Most patients showed symmetric VOR gain deficits on both sides [7]. This is the reason why these patients do not have vertigo since vertigo will only manifest when the vestibular deficit is unilateral. 

Most previous studies on GVT focused on patients with severe BVL that was measured through caloric and rotational tests [23]. When vHIT was used for the evaluation of vestibular function in BVL related to the use of aminoglycosides, the gains were close to our results for group A [24].

The vestibular damage in group A can be considered as “permanent” because it was registered two months after the treatment began and ended. However, the damage in group B can be considered as “temporary” because it was registered for less than a week after the treatment began and practically at the time when the symptoms started. For this reason, the vHIT was repeated one month later in group B (and after changing gentamicin), showing a significant improvement in the gain, and reaching the lower limit of normality on the best side. The physiology of the cell can be recovered once the aminoglycoside is separated from the cell receptor, which is why the vestibular function returns to normal after acute intoxication in some patients [5,25].

This shows that after the early evaluation by the neurotologist and discontinuation of gentamicin and using a non-ototoxic drug (second choice for infective endocarditis protocol) as previously agreed between the cardiologist and the neurotologist, vestibular loss was reduced after one month.

Patients with loss of vestibular function are unable to stabilize vision during head motion toward the affected side, experiencing blurred vision, and they need to make “catch-up” saccades to refixate the gaze [26]. One of the most important symptoms of BVH is oscillopsia [16]. It is well known that vHIT gains in patients with BVH are low [27].

A statistically significant difference was found when comparing the gains in the vHIT of patients who had oscillopsia versus those who did not. Those who suffered from oscillopsia showed gains lower than 0.6 on both sides in the test, defining BVH according to previous studies [16].

Oscillopsia is a very disabling symptom that is directly related to the level of vestibular dysfunction. Therefore, it is very important to prevent patients from exceeding a certain limit in their loss of vestibular function.

## 5. Conclusions

The inclusion of vestibular function evaluation in an infective endocarditis treatment protocol should be recommended, considering the adverse effects of aminoglycosides.

The adverse effects of systemic gentamicin in the inner ear can be controlled by paying attention to the patient’s symptoms as soon as they start and replacing this medication with a non-ototoxic drug.

Bilateral vestibular loss produces very disabling symptoms, such as oscillopsia, which have a significant impact on the patient’s quality of life and therefore must be prevented.

We recommend questioning patients daily about vestibular symptoms and, if they appear, performing an early neurotological evaluation, in order to detect vestibular deficits, and withdrawing gentamicin. Patients must be re-evaluated before discharge and at least one month after completing the treatment.

Prospective randomized studies are needed to clarify the implications of early intervention, including the withdrawal of systemic gentamicin and its association with bilateral vestibular loss. Moreover, this study emphasizes the crucial importance of such studies.

## Figures and Tables

**Table 1:** Main data of the sample.

	Group A	Group B	
Patients	13	16	
Age	58 ± 6	54 ± 7	
Sex	7 men/6 women	9 men/7 women	
Days from treatment to test	70	6	*p* < 0.0001
Days from symptoms to test	65	2	*p* < 0.0001
Days treatment to symptoms	5	4	
Imbalance	31%	69%	*p* = 0.042
Imbalance + dizziness	69%	31%	*p* = 0.042
Oscillopsia	62%	19%	*p* = 0.027
Worst side gain	0.39 ± 0.11	0.64 ± 0.11	*p* < 0.0001
Best side gain	0.44 ± 0.10	0.71 ± 0.12	*p* < 0.0001
Worst side gain after 1 month		0.72 ± 10	
Best side gain after 1 month		0.80 ± 10	
	Oscillopsia	No Oscillopsia	
Worst side gain	0.35 ± 0.07	0.63 ± 0.09	*p* < 0.0001
Best side gain	0.41 ± 0.07	0.70 ± 0.11	*p* < 0.0001

## Data Availability

There are both ethical and legal restrictions on sharing the original study datasets. The electronic health records data cannot be shared publicly because it consists of personal information from which it is difficult to guarantee de-identification (Law 03/2018 from Spanish Government—BOE-A-2018-16673). There is a possibility of deductive disclosure of participants and therefore full data access through a public repository. The original datasets could only be made available under a new data sharing agreement with which includes: (1) commitment to using the data only for research purposes and not to identify any individual participant; (2) a commitment to securing the data using appropriate measures, and (3) a commitment to destroy or return the data after analyses are complete. For more information on data availability restrictions you can contact the ethics committee local IRB CEIm Area de Salud de Salamanca at comite.etico.husa@saludcastillayleon.es. Requests can be made to the corresponding author, who will connect the request to designated IRB representatives, and eventually send the information.
